# Dynamic Scenario of Membrane Binding Process of Kalata B1

**DOI:** 10.1371/journal.pone.0114473

**Published:** 2014-12-04

**Authors:** Wanapinun Nawae, Supa Hannongbua, Marasri Ruengjitchatchawalya

**Affiliations:** 1 Biological Engineering Program, Faculty of Engineering, King Mongkut’s University of Technology Thonburi, Thung Khru, Bangkok, Thailand; 2 Department of Chemistry, Faculty of Science, Kasetsart University, Chatuchak, Bangkok, Thailand; 3 Bioinformatics and Systems Biology program, King Mongkut’s University of Technology Thonburi (Bang Khun Thian), Bang Khun Thian, Bangkok, Thailand; 4 Biotechnology Program, School of Bioresources and Technology, King Mongkut’s University of Technology Thonburi (Bang Khun Thian), Bang Khun Thian, Bangkok, Thailand; University of Queensland, Australia

## Abstract

Kalata B1 (kB1), a cyclotide that has been used in medical applications, displays cytotoxicity related to membrane binding and oligomerization. Our molecular dynamics simulation results demonstrate that Trp19 in loop 5 of both monomeric and tetrameric kB1 is a key residue for initial anchoring in the membrane binding process. This residue also facilitates the formation of kB1 tetramers. Additionally, we elucidate that kB1 preferentially binds to the membrane interfacial zone and is unable to penetrate into the membrane. In particular, significant roles of amino acid residues in loop 5 and loop 6 on the localization of kB1 to this membrane-water interface zone are found. This study reveals the roles of amino acid residues in the bioactivity of kB1, which is information that can be useful for designing new therapeutic cyclotides with less toxicity.

## Introduction

Cyclotides are peptides found in the Rubiaceae, Violaceae, Cucurbitaceae and Apocynaceae plant families [1], [2]. These peptides are characterized by a cyclic cysteine knot motif [3]. The amino acid (AA) sequences of all cyclotides are divided into six loops according to six conserved cysteine residues [1]. Cyclotides display various therapeutic activities such as anti-microbial [4], anti-HIV [5], [6] and anti-cancer [7]. However, their usage as drugs is still far from reality because of their cytotoxicity [1]. Notwithstanding, cyclotides are remarkably stable peptides, and their sequence can be modified without serious effects on their overall folding [2], [8]. The structure of cyclotides is, therefore, one of the most promising scaffolds for therapeutic peptide design generally by integrating the bioactive peptide sequence into the cyclotides sequence [9]–[13]. Cyclotides in the trypsin inhibitor subfamily have received attention from many sequence bioengineering studies [14]–[19]. However, there are few cyclotides in this subfamily [20]. Two other subfamilies are the Bracelet and Möbius subfamilies, which account for approximately 67% and 33% of the total number of discovered cyclotides, respectively [13]. However, cyclotides in the Bracelet subfamily have never been bioengineered [13]. In the Möbius subfamily, only kalata B1 (kB1) has ever been bioengineered [21]–[23] and used as an uterotonic agent by African tribes [24].

**Figure 1:**
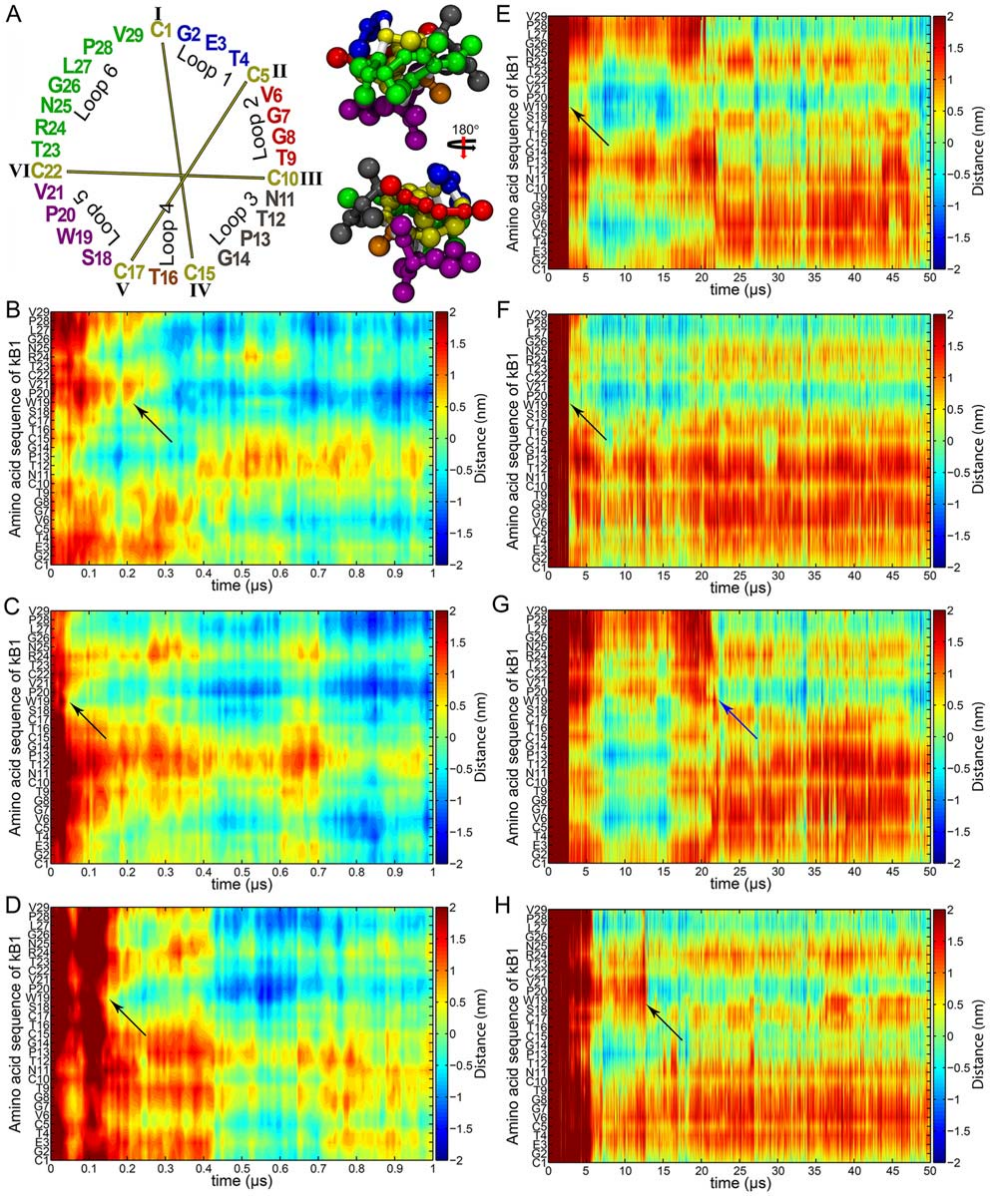
Membrane binding progression of kB1. (A) Sequence and coarse-grained model of kB1 structure. The amino acid (AA) sequences of kB1 and other cyclotides are divided into six loops. Loops 1–6 of kB1 are colored blue, red, grey, orange, violet and green, respectively. Cysteine is shown in yellow and disulfide bonds are presented with yellow lines. The structure of kB1 is shown as a space-filling CPK model. The loop colors are the same as those shown for the sequence. The peptide bond of any AA residue to cysteine is shown in white. The distances of all AA residues relative to the membrane surface of the monomer in the (B) M1, (C) M2 and (D) M3 simulations are presented. The relative distances are shown during 0–1 µs to clearly demonstrate the activity of Trp19 in the membrane binding process of kB1. The distances of all AA residues of kB1 molecules (E) A, (F) B, (G) C and (H) D in the tetramer relative to the membrane surface during the entire simulations are shown. Black arrows show the membrane binding of Trp19. The blue arrow shows the binding of the Trp19 of molecule C to the membrane at approximately 22.3 µs, which was the time that the tetramer completed its membrane binding process.

kB1 is an amphipathic peptide containing 29 AA residues. Based on the hydrophobicity scale used in the Cybase database [20], most of the hydrophilic residues are found in loops 1–4, whereas hydrophobic residues are located in loop 5. Loop 6 of the peptide contains four hydrophilic residues and three terminal hydrophobic residues (Figure 1A). So far, experimental studies have investigated the relationships between AA residues and bioactivities including insecticidal [25], nematocidal [26] and lipid bilayer leaking [27] of kB1 by using the site-directed mutagenesis method. The mechanism of several bioactivities (including its cytotoxicity) of kB1 is related to membrane binding and oligomerization [27]–[29]. The membrane binding of kB1 eventually causes membrane disruption. Previously, we demonstrated that kB1 binds to the membrane in both monomeric and oligomeric forms [29], of which tetramers are one of the major forms of kB1 oligomerization [27]–[29].

Coarse-grained molecular dynamics (CG-MD) simulations is popularly used to study the complex bioactivity of biological macromolecules [30]. Previously, we used CG-MD simulations to study the membrane disruption mechanism of kB1 [29]. CG-MD simulations has also been used to describe the aggregation and membrane disruption of a cyclic antibacterial peptide [31]. The method was also used to identify loops that play roles in the membrane penetration activity of cobra cytotoxic peptides [32]. Another simulation technique, pulling and umbrella sampling simulations, has been used to illustrate the different membrane permeation scenarios of several α-helical peptides [33] and to facilitate the design and screening for effective anti-microbial peptides that display less hemolytic activity [34].

In this study, AA residues that are important for the membrane binding process of kB1 are identified by CG-MD simulations. The pattern of kB1-kB1 interactions in the tetramer is depicted. In addition, we estimated free-energy differences using umbrella sampling [35] to elucidate the binding of kB1 to the membrane interfacial zone [36].

## Results

### kB1 Trp19 is a key residue in the membrane binding process

Three simulations each were performed for the monomer (M1–M3) and the tetramer (T1–T3) (Table S1). In each simulation of the monomer, which was conducted for 10 µs, a kB1 molecule was added to the system with a random orientation, and the center of mass (COM) of the peptide was located at approximately 2.0 nm above the membrane surface. To elucidate the activity and interplay of amino acid (AA) residues during the membrane binding process, distances from every AA residue of kB1 molecules to the membrane surface were measured as a function of time (Figure 1B–1H). In the M1 system, a group of AA residues in loop 3 including Asn11, Thr12 and Pro13 was the first group that interacted with the membrane surface (Figure 1B). Then, Pro13 bound to the hydrophobic region of the membrane and pulled other residues in loop 3; Thr16 in loop 4; and Thr23, Arg24 and Asn25 in loop 6 into the polar head region of the membrane. However, shortly afterwards, Trp19 in loop 5 bound to the membrane. Its binding changed the orientation of kB1 by inducing the membrane binding of all AA residues in loop 5; Leu27, Pro28 and Val29 in loop 6; and Val6 in loop 2. An explicit orientation change was not detected afterwards, indicating the final membrane-bound orientation of kB1. This membrane-bound orientation of kB1 corresponded with the structure reported in a nuclear magnetic resonance (NMR) spectroscopy study [37]. With the M2 system, the orientation change of the kB1 molecule was hardly detected (Figure 1C). This is because the hydrophobic residues in loops 5 and 6 were the first group that bound to the membrane. The final membrane-bound orientation of kB1 obtained in this system was the same as that observed in the M1 system. In the M3 system, we observed AA residues in loops 1, 2 and 3 approaching the membrane during the initial period of the simulation (during 0.05–0.08 µs in Figure 1D). However, during this time period, the peptide did not bind to the membrane but moved away from the surface of the membrane. Nevertheless, shortly afterwards, Trp19 bound to the membrane and promoted the final membrane-bound orientation of kB1.

For the tetramer, each simulation was conducted for 20 µs because the initial position of each kB1 molecule, namely molecules A, B, C and D (Figure S1 and S2), was approximately 6.0 nm above the membrane surface. Based on positions of the peptides, the tetramer was formed in water. However, we observed that the kB1 tetramer was able to bind to the membrane within 20 µs only in the T1 simulation. Thus, only the T1 simulations was used to illustrate formation and membrane binding processes of the kB1 tetramer. The membrane binding of the tetramer began at 2.8 µs when the Trp19 residues of molecules A and B were haphazardly exposed to the water while they were approaching the membrane (Figure 1E, 1F and S3). Trp19, Pro20 and Val21 in loop5 of molecules A and B then bound to the membrane. Subsequently, Trp19 of molecule C bound to the membrane (Figure 1G). At 12.8 µs, Trp19 of molecule D suddenly inserted into the membrane (Figure 1H). This pushed Trp19 of molecule C away from the membrane. This Trp19 did not re-insert into the membrane within 20 µs. We then decided to extend the simulation to 50 µs and observed that Trp19 of molecule C re-inserted into the membrane at approximately 22.3 µs (blue arrow in Figure 1G). After the membrane binding of this Trp19, no significant changes to the membrane-bound orientation of the kB1 molecules in the tetramer were detected (during 23–50 µs in Figure 1E–1H). This marked the completion of the membrane binding process of the tetramer. The residues of all kB1 molecules in the tetramer that bound to the hydrophobic region of the membrane were the same as those of the monomer except Val6 in loop 2.

The membrane binding process of the kB1 monomer in each simulation was found to be completed within 0.5 µs. In contrast, kB1 molecules in the tetramer did not bind to the membrane during the 0–2.7 µs interval, although there were several times that they approached the membrane surface (Figure S4). The tetramer began its membrane binding process at 2.7 µs and completed the process at approximately 22.3 µs. Moreover, the tetramers in the T2 and T3 simulations were unable to bind to the membrane within 20 µs. These results indicate that, compared to the monomer, it was significantly harder for the tetramer to bind to the membrane.

### Importance of Trp19-Trp19 interactions in kB1 tetramer

**Figure 2:**
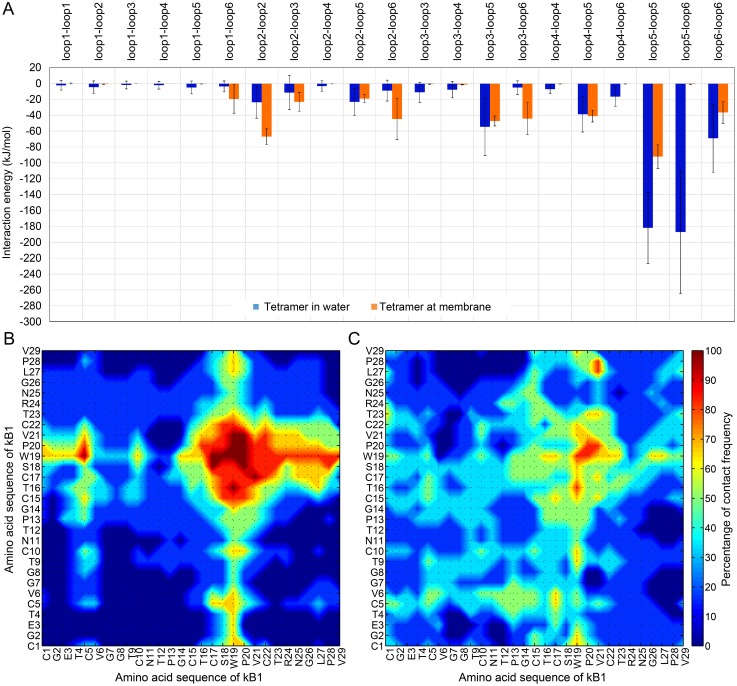
Pattern of kB1-kB1 interaction. (A) The intermolecular loop-to-loop interaction energies of the tetramer in water (blue bar) and in its membrane-bound state (orange bar) are shown. The interaction energies were averaged across 22.3–5.0 µs. The error bar represents the standard deviation of the average values. Matrices representing the contact frequencies for the tetramer in (B) water and (C) in its membrane-bound state are shown. The contact frequency for each AA residue pair of the tetramer in water and in its membrane-bound state were measured from 0–2.6 and 22.4–5.0 µs, respectively.

To understand the complex membrane binding process of the tetramer, the kB1-kB1 interaction pattern was explored through investigation of the intermolecular loop-to-loop interaction energy and the contact frequency of every pair of AA residues in the tetramer (see method). In water, the total kB1-kB1 interaction energy was ca. −669±94 kJ/mol. We observed that the intermolecular loop5-loop5 interaction energy accounted for 28% of this total interaction energy (Figure 2A). The contact-frequency analysis indicated that AA residues in loop 5 of one kB1 molecule in the tetramer contacted the same residues of other kB1 molecules with a frequency of 80–100% (Figure 2B). The results also showed that intermolecular Trp19-Trp19 contact was the core of the loop5-loop5 contact and displayed a contact frequency of 100%. In addition, the simulation snapshot showed the pairing between side chains of the Trp19 residues of molecules A and B and between those of molecules C and D (Figure S1). AA residues in loop 5 also contacted hydrophobic residues in loop 6 with a frequency of 60–80%. Their intermolecular interaction energy was 27% of the total interaction energy. The contact frequencies of other loop pairs were less than 60%, and their interaction energies were lower than 10% of the total interaction energy. The results indicate that hydrophobic residues in loop 5, especially Trp19, were responsible for tetramer formation. Accordingly, an NMR spectroscopy experiment indicated that kalata B2 (kB2) utilizes its hydrophobic residues to form tetramers and octamers in solution [38].

**Figure 3:**
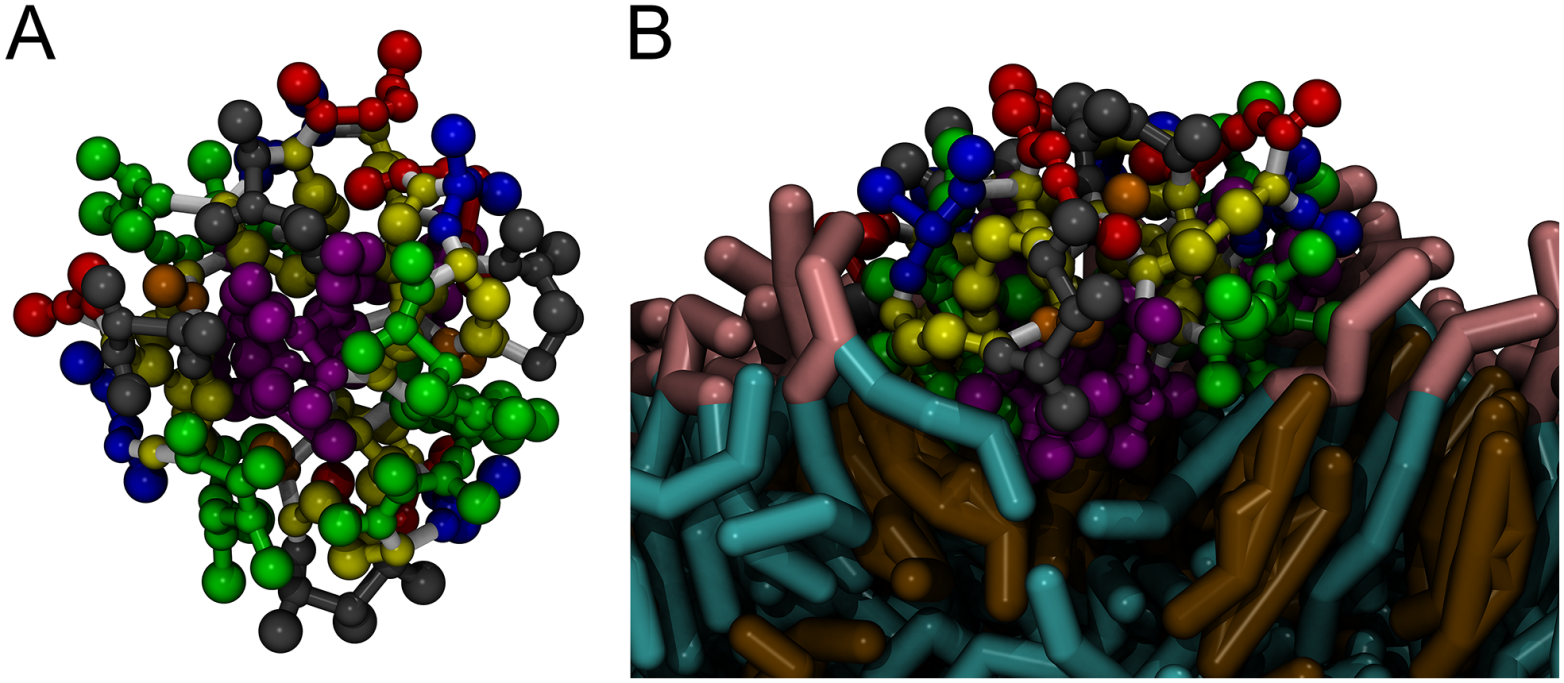
Different conformation of kB1 tetramer. (A) Top view of the tetramer in water. (B) Side view of the tetramer in its membrane-bound state. The structure of kB1 is shown as a CPK model with the same colors as in Figure 1A. Lipid molecules are represented as licorice models. The polar heads and hydrophobic tails of DPPC and DUPC lipids are shown in pink and cyan, respectively. Cholesterol molecules are presented in brown. For clarity of the Figure, water molecules are not shown.

Once the tetramer successfully bound to the membrane, the total kB1-kB1 and the intermolecular loop5-loop5 interactions energies were ca. −439±43 kJ/mol and −22±4 kJ/mol, respectively. These results indicate that the kB1-kB1 and loop5-loop5 interactions of the tetramer at membrane bound state were less favorable than the same interactions of the tetramer in water (Figure 2A). The intermolecular contact frequencies between AA residues in loop 5 were 60–85% (Figure 2C). However, the loop5-loop5 interaction was still the major interaction between kB1 molecules in the tetramer in the membrane-bound state. In addition, the Trp19-Trp19 contact frequency remained the highest among all residue-to-residue contacts, and the pairing between side chains of Trp19 residues was also observed (Figure S2). More interestingly, the intermolecular loop5-loop6 interaction energy of the tetramer was 99% higher in the membrane-bound state than it was in water (Figure 2A). In contrast, the intermolecular interaction energies of loop 6 with loop 1, 2 and 3 were 80%, 80% and 88% lower, respectively, than those of the same interactions in water. Another interesting interaction is the intermolecular loop2-loop2 interaction because its energy was found to be 64% lower than that of the tetramer in the water. We also observed that hydrophobic residues in loop 5 and loop 6 were buried (but not completely) in the center of the tetramer in water (Figure 3A). By contrast, hydrophilic residues in loops 1, 2, 3 and 6 were exposed to water and hardly interacted with each other. Once the tetramer bound with the membrane, the hydrophobic residues expanded from the center of the tetramer (while conserving the loop 5-loop 5 interaction) and bound to the hydrophobic region of the membrane (Figure 3B). This configuration change allowed hydrophilic residues in loops 1, 2, 3 and 6 to interact with each other.

### Preferential binding of kB1 to the membrane interfacial zone

To illustrate the favorable location of kB1 on the membrane, positions of the peptides relative to the lo domain were investigated. For the monomer, approximately 92% and 8% of the time between 1–50 µs accounted for the time that kB1 located in the ld domain and at the lipid domains interface, respectively. Similarly, approximately 90% and 10% of the time after all kB1 molecules in the tetramer bound to the membrane (between 23–50 µs) were used by the peptides to locate in the ld domain and at the lipid domains interface. For both simulations, kB1 did not locate in the lo domain. These results indicate that kB1 both in monomeric and tetrameric form preferred binding to the ld domain.

To explore the possibility that kB1 penetrates the membrane, pulling and umbrella sampling simulations were performed. The starting configurations for the pulling simulations were selected during last 10 µs of the monomer and tetramer simulations because during this period of time kB1 located in their favorable binding location, the ld domain. In the pulling simulations, both the kB1 monomer and tetramer were pulled from the water to the COM of the membrane. Thereafter, umbrella sampling simulations were conducted to estimate free energy changes as a function of the kB1 positions relative to the COM of the membrane.

**Figure 4:**
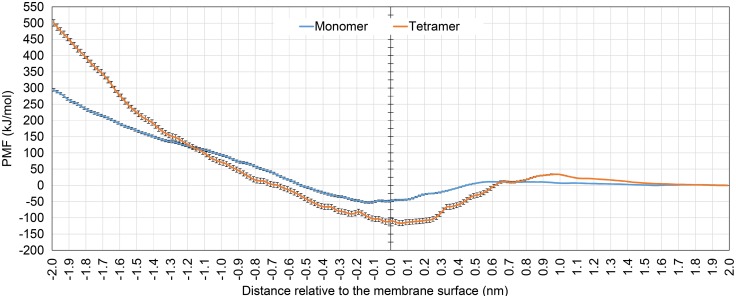
Potential of kB1 to penetrate the membrane. Mean force potentials for monomeric (blue line) and tetrameric (orange line) kB1 transferring from water (2.0 nm) across the membrane surface (0.0 nm) to the COM of the membrane (−2.0 nm) are shown. Error bars were estimated using bootstrap analysis.

For the monomer, we observed an energy barrier of 11±1 kJ/mol (compared to the potential of mean force (PMF) of kB1 in the water phase) when the COM of the peptide was 0.6 nm above the membrane surface (Figure 4), possibly because hydrophobic residues of the kB1 molecule contacted the polar head of the membrane. When the peptide was located at approximately 0.1–0.2 nm below the membrane surface, the free energy difference was −53±3 kJ/mol, which was the lowest energy observed. Correspondingly, the average distance from the monomer to the COM of the membrane was 1.9±0.2 nm (Figure S5). When the monomer was located at the COM of the membrane (2.0 nm below the membrane surface), the maximum energy difference was 294±4 kJ/mol. On the other hand, a free energy barrier of 35±2 kJ/mol was detected for the tetramer when its COM was approximately 1.0 nm above the membrane surface (Figure 4). The lowest energy difference of this simulation was −116±8 kJ/mol when the tetramer was approximately 0.1 nm above the membrane surface. Accordingly, the relative distances of the kB1 molecules in the tetramer were in the range of 1.9–2.0 nm with the standard deviation between 0.2–0.3 nm (Figure S5). The maximum energy difference of 501±10 kJ/mol was observed when the tetramer was located at the membrane center. These results reveal that kB1 molecules preferred binding to the interfacial zone of the membrane. In contrast, the hydrophobic region of the membrane was an unfavorable environment for kB1 where the center of the membrane was the most unfavorable location (Figure 4).

**Figure 5:**
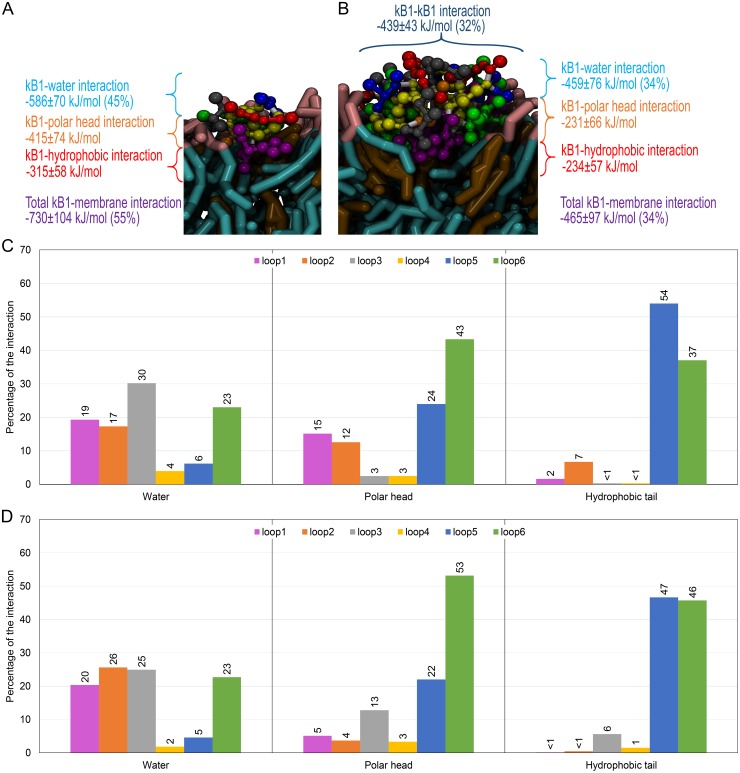
Interaction of monomeric and tetrameric kB1 in membrane-bound state. Average interaction energies of (A) monomeric and (B) tetrameric kB1 in the membrane-bound state are shown. In each parenthesis, the percentage of the interaction energy relative to the total interaction energy is presented. The structures are shown as the same model as in Figure 3. The interaction energy of each loop with water and with the membrane’s polar head and hydrophobic tail for (C) monomeric and (D) tetrameric kB1 are shown as percentages of the total interaction energy.

To understand the binding of kB1 to the membrane interfacial zone, the interaction energies of the peptide with both the membrane and the water were measured. We observed that 45% and 55% of the total monomer interaction energy was due to its interaction with the water and the membrane, respectively (Figure 5A and S6). For the tetramer, 34%, 34% and 32% of the total interaction energy accounted for the kB1-water, kB1-membrane and kB1-kB1 interactions, respectively (Figure 5B). These results indicate that almost a half of kB1’s surface favored interaction with water while the other half preferred to bind to the membrane. This interaction pattern explains the preference of kB1 for binding to the membrane interfacial zone.

The interaction energies of each loop and each AA residue of kB1 with water and with the polar head and hydrophobic tail of the membrane were also measured (Figure 5C and 5D). The interaction energy of each loop with water was presented comparatively as a percentage of the total interaction energy between all kB1 loops and water. The same representation was also applied to the interaction energy between each loop and the polar head and hydrophobic tail of the membrane. For the interaction with water, loops 1, 2, 3 and 6 of both the monomer and the tetramer displayed relatively strong interactions. The AA residues that are important for the interaction with water were Glu3 in loop 1, Thr9 in loop 2, Thr12 and Asn11 in loop 3 and Arg24 in loop 6 (Table S2). Regarding the interaction with the membrane, the interaction energies of the monomer with the polar head and the hydrophobic tail were lower than those of the tetramer (Figure 5A and 5B). This is because kB1 molecules in the tetramer formed intermolecular loop5-loop5 interactions. As a result, some AA residues in loop 5 could interact with the membrane only partially or not at all. The results also showed that, in both the monomer and tetramer, loop 6 displayed the strongest interaction with the polar head of the membrane and Arg24 was the most important residue (Table S2). Loop 5 of both the monomer and the tetramer displayed a moderate interaction (compared with loop 6) with the polar head of the membrane, in which Trp19 was the strongest interacting residue (Figure 5C and 5D). Loop 5 and loop 6 are two major loops that were found to interact with the hydrophobic tail. The residues that strongly interacted with the hydrophobic tail were Trp19, Pro20 and Val21 in loop 5 and Leu27, Pro28 and Val29 in loop 6 (Table S2). Among these residues, Trp19 displayed lowest interaction energy.

## Discussion

We have identified AA residues of kB1 that play significant roles in the process of membrane binding and their tetramer formation, inferring the relationship between the AA sequence and the bioactivity. Our MD simulations revealed that Trp19-Val21 in loop 5 and Leu27-Val29 in loop 6 of kB1 bind to the hydrophobic region of the membrane while hydrophilic residues in loop 6 (Thr23-Gly26) locate to the polar head region of the membrane. Additionally, our results demonstrated that Trp19 is a key residue that facilitates the membrane binding process of kB1. The tryptophan is known as a membrane-anchoring residue in several membrane-binding proteins in regard to its containing an indole group in the side chain that can form a hydrogen bond with polar head atoms of the membrane while its aromatic ring prefers hydrophobic interactions [39], [40]. Accordingly, an experimental study indicated that kB1 (monomer) bind to the membrane via hydrophobic residues in loops 5 and 6 [37]. Moreover, an alanine-scanning mutagenesis study indicated that Trp19 and Pro20 are important residues for the lytic activity of kB1 [27].

The patterns of interaction between kB1 molecules in the tetramer indicated that the intermolecular loop5-loop5 interaction holds the kB1 molecules together whether the tetramer is in water or at membrane-bound state. Among the residues in loop 5, Trp19 is the most important AA residue. In addition, the tetramer in water and at membrane-bound state adopts different conformations (Figure 3) to balance their hydrophobic and hydrophilic interactions in different environments of water and membrane. The results also illustrate that it is difficult for the tetramer to bind to the membrane. This is because the hydrophobic residues in loop 5, especially Trp19 which are strongly required for the membrane binding of kB1, are buried in the center of the tetramer in water (Figure 3 and S3). Consistently, the oligomerization of kB2 in solution, which is facilitated by the interactions of its hydrophobic residues, might block the membrane disruption activity of this peptide [38].

The tetramer can bind to the membrane only when Trp19 is exposed to the water and, at the same time, is located near the membrane surface. However, the possibility of this occurrence is low. Consistently, we have previously observed that, instead of directly bind to the membrane, kB1 oligomers in the water bind to kB1 monomers that successfully bind to the membrane [29]. Thereby, kB1 oligomers can quickly bind to the membrane because they were localized near the membrane surface and the exposure of Trp19 to the membrane surface might be facilitated.

In addition, our results in this study and [29] demonstrated that kB1 molecules whether in the monomeric, tetrameric or oligomeric forms do not penetrate into the membrane. The PMF profiles obtained in this study display the same pattern as that of a helix peptide that binds to the membrane interfacial zone [33]. The minimum and maximum PMF values were observed when the peptides locate at the membrane surface and the membrane center, respectively. This is because almost a half of the peptides surface is occupied by the hydrophobic residues whereas the other half is occupied by the hydrophilic residues. The interaction energies of kB1 with water and the membrane (Figure 5) indicate that loops 5 and 6 are important loops that anchor kB1 at the interfacial zone. Loop 6 is the longest loop of kB1 and AA residues in this loop display relatively strong interactions with water and with the polar head and hydrophobic tail of the membrane (Figure 5C and 5D), which are the overall components of the interfacial zone. Interestingly, Arg24, which contains a long charged side chain and is conserved in most cyclotides of the Möbius subfamily [20], [41], is found to strongly interact with water and the polar head of the membrane. Additionally, the results of Leu27, Pro28 and Val29 in loop 6 that bind to the hydrophobic region of the membrane indicate that the distribution of the hydrophilic and hydrophobic residues on the kB1 surface is important not only for the membrane-bound orientation of kB1 but also for the positioning of this peptide in the membrane interfacial zone.

**Figure 6:**
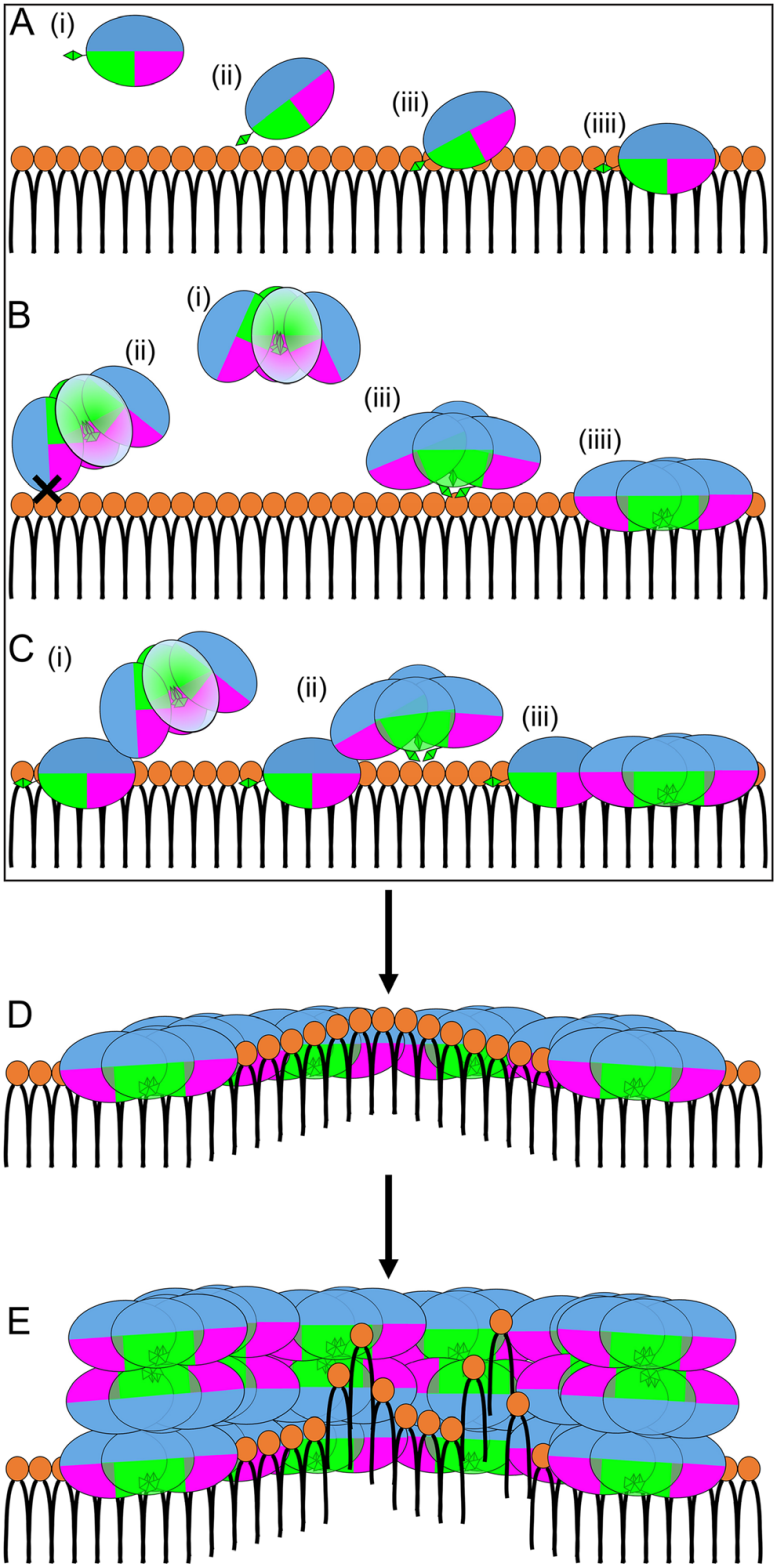
Mechanism of membrane binding and disruption by kB1. (A) The membrane binding mechanism of monomeric kB1. (i) Before the membrane binding process, Trp19 (extended green trapezoid) is exposed to the water. (ii) Membrane binding begins when Trp19 binds to the membrane. (iii) Hydrophobic residues in loop 5 (green patch) then bind to the membrane. (iiii) Next, hydrophobic residues in loops 5 and 6 (pink patch) bind to the hydrophobic region of the membrane while hydrophilic residues in loops 1, 2, 3 and 6 (blue patch) localize to water and the polar head region of the membrane. (B) The membrane binding mechanism of tetrameric kB1. (i) Loop 5 of one kB1 molecule interacts with loop 5 of other kB1 molecules to form a tetramer in the water. (ii) The tetramer cannot bind to the membrane when hydrophilic residues in loops 1, 2, 3 and 6 or hydrophobic residues in loop 6 reach the membrane. (iii) The tetramer binds to the membrane when Trp19 is exposed to water and is proximal to the membrane surface. (iiii) Hydrophobic residues in loop 5 then bind to the membrane. All hydrophobic residues in loop 6 then expand from the center of the tetramer and bind to the membrane. (C) The membrane binding of kB1 as a tower like cluster [29]. (i) Tetramer in the water bind to a monomer that is successfully bound to the membrane. (ii) In the tower-like cluster, the tetramer is held near the membrane surface. (iii) A wall-like cluster [29] is formed. (D) kB1 molecules do not penetrate the membrane but form a channel on the membrane surface and induce positive membrane curvature [29]. (E) At high kB1 concentrations, lipid molecules are extracted from the membrane to the channel inside the kB1 cluster [29]. All panels are shown in side view. Only half of the kB1 channel is shown in (D) and (E). Polar heads of lipids are shown as orange circles while hydrophobic tails are shown as black lines.

Based on our results from this and previous [29] studies, we propose a more complete view of the mechanism of the membrane binding and disruption by kB1 (Figure 6). The peptide binds to the membrane via three different ways (Figure 6A–C). First, kB1 in monomeric form quickly binds to the membrane (Figure 6A). Second, kB1 binds to the membrane with difficulty when it is in tetrameric form (Figure 6B). Once such kB1 molecules successfully bind to the membrane, they remain in tetrameric form because the intermolecular loop5-loop5 interaction is sustained (Figure 2C and 3B). Third, instead of binding directly to the membrane (which takes a long time), tetramers in the water bind to monomers and oligomers that already bound to the membrane and form tower-like clusters [29] (Figure 6C). In the tower-like clusters, tetramers are localized near the membrane surface, thereby increasing the likelihood that they will bind to the membrane. Then, the kB1 molecules (at 48–120 molecules per 1,000 lipids molecules) begin disrupting the membrane by inducing positive membrane curvature while they are still located at the membrane-water interface area [29] (Figure 6D). Finally, when the concentration of the peptide is raised up to 350 molecules, lipid molecules in the curved area of the membrane are extracted to a channel inside the kB1 cluster [29] (Figure 6E).

In summary, this study describes the role and interplay of AA residues in two important processes, membrane binding and oligomerization, of several bioactivities of kB1. This approach allowed us to identify the important loops (loop 5 and loop 6) and the most important AA residue (Trp19) in the mechanism. The results may also guide the rational bioengineering of therapeutics cyclotides, particularly those in the Möbius subfamily.

## Methods

### Molecular dynamics simulations

All simulations were carried out using the GROMACS software [42], [43] version 4.5. The MARTINI CG force field [44]–[46] was used to present the intramolecular and intermolecular interactions of molecules in the system. All MD simulations were performed under the NPT ensemble where number of particles, pressure, and temperature were kept constant. The temperature was independently coupled for each molecule type in the system to 310 K using the Berendsen thermostat [47]. The pressure was coupled to 1 atm with a compressibility of 4.5×10^–5^ bar^–1^ using the Berendsen barostat [47]. The coupled pressure was conducted with a semi-isotropic scheme where the x, y plane and the z direction were coupled separately to obtain a tensionless membrane. A periodic boundary condition with standard non-bonded interaction criteria was applied to all systems. The leap frog integration algorithm with a time step of 20 femtoseconds was used to solve the motion equation. The van der Waals and electrostatic interactions cut-off were set to 1.2 nm. The visual molecular dynamics (VMD) software [48] was used to visualize and analyze simulations results.

### Preparation of a CG model of kB1

A CG model of the kB1 molecule was prepared based on the solution structure of kB1 obtained from the protein data bank (PDB code: 1NB1 [49]). The “atom2cg_v2.1.awk” script provided on the MARTINI website (http://md.chem.rug.nl/cgmartini/images/tools/atom2cg_v2.1.awk; latest access 30 July 2014) was used to convert the atomistic coordinates to CG coordinates. The MARTINI website also provided the “seq2itp.pl” script (http://md.chem.rug.nl/cgmartini/images/tools/seq2itp/seq2itp.pl; latest access 30 July 2014) that was used to generate the GROMACS topology file. This file contains the parameters associated with bonds between CG atoms and the folding of the CG model of the kB1 molecule. However, the generated topology does not contain parameters to describe a peptide bond between the N and C termini of the cyclic peptide. Based on the kB1 sequence, the parameters of the peptide bond between Cys1 (the C terminus) and Val29 (the N terminus) were obtained from the bond parameters between Val21 and Cys22. Standard protonation states at neutral pH have been assigned to all amino acid residues, resulting with the total charge of zero.

### Preparation of the membrane model

To mimic a raft containing membrane which is an updated view of biological membrane [50], the membrane model used in this study is a heterogeneous membrane that contains liquid ordered (lo) and liquid disordered (ld) domains. Following the successful CG-MD simulations of the heterogeneous membrane models [50], the membrane model was composed of diundecanoyl-phosphatidylcholine (DUPC), dipalmitoyl-phosphatidylcholine (DPPC) and cholesterol (CHOL). DPPC represents saturated lipid and interacts preferentially with CHOL which can increase the compressibility of nearby lipids [51]. Therefore, the packing between DPPC molecules is very tight [50]. In contrast, the packing between DUPC molecules, which represents unsaturated lipid, is loose. Because of these different packing abilities, the lo domain enriched with CHOL and DPPC is phase separated from the ld domain, which contains mostly DUPC [50]. The membrane model preparation method used in [29] was applied in this study. The DUPC, DPPC and CHOL ratio is 0.07∶0.62∶0.31, which was translated from the lipid ratio obtained from an experimental study [52]. Each lipid molecule was hydrated by 6 water beads [53]. The starting structure of the membrane model was prepared using our in-house software called the automated membrane generator. The program automatically generated the heterogeneous membrane model by constructing the lipid domains of DPPC and CHOL, which were surrounded by DUPC and CHOL. To relax the interaction between lipid molecules, a simulation of 4 µs was conducted for the pure membrane.

### Simulations system setup

Six simulations were performed to study the membrane binding of kB1 monomers, kB1 tetramer formation and tetramer membrane binding (Table S1). To setup all the simulations systems, the membrane model at 4 µs (see above) was used. Water beads were removed from the membrane system. After that, kB1 molecules were added to the systems with a random orientation. The initial distance from the COM of the peptide in each monomer simulation to the COM of the membrane surface was approximately 2.0 nm while the initial distance of each kB1 molecule in each tetramer simulation was approximately 6.0 nm. 2,400 water beads were then added into the system. The size of all simulations boxes was approximately 16×16×16 nm^3^.

### Potential of Mean Force Calculations

The molecular coordinates that would be used to prepare initially configurations for the pulling simulations of the monomer and tetramer were randomly selected during 40–50 µs (Figure S7A). The peptides were pulled for 2 nm from the membrane surface toward the water using a harmonic potential with a constant force of 1,500 kJ/(mol nm^2^). Each system was then equilibrated for 1 ns by constraining the peptide position. The equilibrated systems were then used as initial configurations for PMF calculations (Figure S7B). The peptides were pulled along the Z axis (normal axis of the membrane) from the water (2 nm) cross the membrane surface (0 nm) to the COM of the membrane (−2 nm) using a constant force of 1,500 kJ/(mol nm^2^). For this 4 nm of the peptides moving, the molecular coordinates of the system were extracted every 0.1 nm. Each molecular coordinate is called an umbrella sampling window. Each window was simulated for 100 ns wherein the COM of the peptides was restrained by a bias potential of 1,500 kJ/(mol nm^2^). The unbiased potential was then calculated using weighted histogram analysis method (WHAM) [54], [55] providing PMF as a function of the distances from the COM of kB1 to the COM of the membrane. To obtain a good overlap of the histogram and hence good PMF profiles, there were 43 sampling windows for the monomer including 41 windows from the 0.1 nm spacing from −2 to 2 nm and 2 additional windows. For the tetramer, there were 45 total sampling windows including 41 windows from the 0.1 nm spacing and 4 additional windows. The statistical errors were estimated using bootstrap analysis [55]. The number of bootstraps was set to 100. The PMF profile obtained from the coarse-grained simulations was validated by comparing to the PMF profile calculated using atomistic (with united atom lipids molecules) simulations. Figure S8 shows that the PMF profiles obtained from these two simulations are very similar indicating the reliability of the MARTINI CG force field [34].

### Contact frequency analysis

The existence of contact between two AA residues was determined by their distance from each other. Initially, the minimum distance between each AA residue pair for every pair of kB1 molecules in the tetramer was measured. A 116 (4 kB1 molecules×29 residues)×116 replica-symmetry matrix that represents the distance values was obtained (Figure S9). We then simplified this complex matrix by translating the distance values to a new representation, e.g. contact and non-contact. If the distance between two AA residues was lower than or equal to 1.2 nm (the short range interaction cut-off), these two AA residues were determined to be in contact with each other. Because there were six pairs of kB1 molecules, the contact of a residue pair was summarized as a frequency. For instance, the frequency of Cys1-Cys1 contact is 2 (Figure S9, magenta dot). On the other hand, the frequency of Cys1-Cys1 non-contact is 4 (Figure S9, blue dot). Contact is presented relatively as a percentage of the total frequency obtained from the following equation:
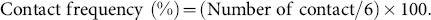



Therefore, the Cys1-Cys1 contact frequency in this example is ca. 33%. This procedure was repeated for all pairs of AA residues. Thereby, the 116×116 matrix was reduced to 29×29 matrix (Figure 2B and 2C).

### Interaction energy measurement

The interaction energies of kB1 molecules in the tetramer with water and with the polar head and hydrophobic tail of the membrane were measured from 23–50 µs. This is because the tetramer successfully bound to the membrane at 23 µs. For the monomer, the M1 simulations was extended from 10 µs to 50 µs and the interaction energies were measured from 23–50 µs for an equivalent comparison with the tetramer.

## Supporting Information

Figure S1 **Pairing between side chains of Trp19 residues of kB1 tetramer in water.** Molecules of kB1 are shown as transparent surface models. Molecules A–D are colored green, cyan, grey and orange, respectively. Trp19 is shown as a CPK model with the same color as the molecules.
(TIF)

Figure S2 **Pairing between side chains of Trp19 residues of kB1 tetramer in membrane-bound state.** Molecules of kB1 are shown as transparent surface models. Molecules A–D are colored green, cyan, grey and orange, respectively. Trp19 is shown as a CPK model with the same color as the molecules. DUPC, DPPC and CHOL are represented as licorice models in blue, red and yellow, respectively. For clarity, water molecules are not shown.
(TIF)

Figure S3 **Exposure of AA residues to water.** The solvent-accessible surface areas of each AA residue of the M1 monomer and all molecules in the tetramer are shown as a function of time across 1–3 µs.
(TIF)

Figure S4 **Approach of the tetramer to the membrane.** The minimum distances of kB1 molecules in the tetramer relative to the membrane surface are shown. We assumed that the tetramer had approached the membrane when the distances were lower than 1.5 nm because at the same distance range the monomer can bind to the membrane. For instance, the distance to the membrane surface from the COM of Gly8 of molecule A at ca. 1.7 µs, Gly8 and Val6 of molecule B at ca. 1.4 µs and Glu3 and Gly7 of molecule D at ca. 1.7 µs was approximately 0.9, 0.7 and 0.7 nm, respectively.
(TIF)

Figure S5 **Position of kB1.** Distances from the COM of monomeric kB1 (from the M1 simulations) and tetrameric kB1 to the COM of the membrane are shown as a function of time. Distances from the membrane surface and glycerol groups are also presented. The offset table shows the average distances and standard deviations from 23–50 µs.
(TIF)

Figure S6 **Interactions of kB1 at membrane-bound state.** Interaction energies of (A) monomeric and (B) tetrameric kB1 as a function of time are shown respectively.
(TIF)

Figure S7 **Configurations for pulling simulations.** (A) Top view of the molecular coordinates selected from the monomer and the tetramer simulations for preparing initial configurations of the pulling simulations are shown. (B) Side view of initial configurations of the monomer and the tetramer used for PMF calculations are shown. DUPC, DPPC and CHOL are represented as licorice models in blue, red and yellow, respectively. The monomer is shown as a green surface model. Molecules A–D are shown as green, cyan, grey and orange surface models, respectively. Water is not shown for clarity.
(TIF)

Figure S8 **Validation of potentials of mean force profile.** Mean force potentials for transferring atomistic (blue line) and coarse-grained (orange line) models of the monomer from the membrane surface (0.0 nm) to the COM of the membrane (−2.0 nm) are shown. Error bars were estimated using bootstrap analysis.
(TIF)

Figure S9 **The minimum residue-to-residue distances matrix.** The distances are shown in a grey scale where the shortest distance (0 nm) is shown as white. As described in the methods, magenta dots represent Cys1-Cys1 contact while blue dots represent Cys1-Cys1 non-contact.
(TIF)

Table S1 Details of the monomeric and tetrameric simulations systems.
(PDF)

Table S2 **Average interaction energy of each AA residue of kB1 in the membrane-bound state.** Interaction energies each AA residue with water and with the polar head and hydrophobic tail of the membrane were calculated in GROMACS program based on MARTINI CG force field. The reported interaction energies were extracted from the GROMACS energy files. Standard deviations of the average interaction energies are presented.
(PDF)

## References

[1] Daly NL, Rosengren KJ, Craik DJ (2009) Discovery, structure and biological activities of cyclotides. Adv Drug Deliv Rev 61:918–930. 19470399 10.1016/j.addr.2009.05.003

[2] Craik DJ (2010) Discovery and applications of the plant cyclotides. Toxicon 56:1092–1102. 20219513 10.1016/j.toxicon.2010.02.021

[3] Craik DJ, Daly NL, Bond T, Waine C (1999) Plant cyclotides: A unique family of cyclic and knotted proteins that defines the cyclic cystine knot structural motif. J Biol Chem 294:1327–1336. 10.1006/jmbi.1999.3383 10600388

[4] Tam JP, Lu YA, Yang JL, Chiu KW (1999) An unusual structural motif of antimicrobial peptides containing end-to-end macrocycle and cystine-knot disulfides. Proc Natl Acad Sci USA 96:8913–8918. 10430870 10.1073/pnas.96.16.8913 PMC17707

[5] Wang CK, Colgrave ML, Gustafson KR, Ireland DC, Goransson U, et al (2008) Anti-HIV cyclotides from the Chinese medicinal herb Viola yedoensis. J Nat Prod 71:47–52. 18081258 10.1021/np070393g PMC6327322

[6] Daly NL, Gustafson KR, Craik DJ (2004) The role of the cyclic peptide backbone in the anti-HIV activity of the cyclotide kalata B1. FEBS Lett 574:69–72. 15358541 10.1016/j.febslet.2004.08.007

[7] Gerlach SL, Rathinakumar R, Chakravarty G, Goransson U, Wimley WC, et al (2010) Anticancer and chemosensitizing abilities of cycloviolacin O2 from Viola odorata and psyle cyclotides from Psychotria leptothyrsa. Biopolymers 94:617–625. 20564026 10.1002/bip.21435

[8] Clark RJ, Daly NL, Craik DJ (2006) Structural plasticity of the cyclic-cystine-knot framework: implications for biological activity and drug design. Biochem J 394:85–93. 16300479 10.1042/BJ20051691 PMC1386006

[9] Garcia AE, Camarero JA (2010) Biological activities of natural and engineered cyclotides, a novel molecular scaffold for peptide-based therapeutics. Curr Mol Pharmacol 3:153–163. 20858197 10.2174/1874467211003030153 PMC3328131

[10] Jagadish K, Camarero JA (2010) Cyclotides, a promising molecular scaffold for peptide-based therapeutics. Biopolymers 94:611–616. 20564025 10.1002/bip.21433 PMC3000894

[11] Henriques ST, Craik DJ (2010) Cyclotides as templates in drug design. Drug Discov Today 15:57–64. 19878736 10.1016/j.drudis.2009.10.007

[12] Gould A, Ji Y, Aboye TL, Camarero JA (2011) Cyclotides, a novel ultrastable polypeptide scaffold for drug discovery. Curr Pharm Des 17:4294–4307. 22204428 10.2174/138161211798999438 PMC3330703

[13] Poth AG, Chan LY, Craik DJ (2013) Cyclotides as grafting frameworks for protein engineering and drug design applications. Biopolymers 100:480–491. 23893608 10.1002/bip.22284

[14] Thongyoo P, Roque-Rosell N, Leatherbarrow RJ, Tate EW (2008) Chemical and biomimetic total syntheses of natural and engineered MCoTI cyclotides. Org Biomol Chem 6:1462–1470. 18385853 10.1039/b801667d

[15] Thongyoo P, Bonomelli C, Leatherbarrow RJ, Tate EW (2009) Potent inhibitors of beta-tryptase and human leukocyte elastase based on the MCoTI-II scaffold. J Med Chem 52:6197–6200. 19772295 10.1021/jm901233u

[16] Sommerhoff CP, Avrutina O, Schmoldt HU, Gabrijelcic-Geiger D, Diederichsen U, et al (2010) Engineered cystine knot miniproteins as potent inhibitors of human mast cell tryptase beta. J Mol Biol 395:167–175. 19852971 10.1016/j.jmb.2009.10.028

[17] Chan LY, Gunasekera S, Henriques ST, Worth NF, Le SJ, et al (2011) Engineering pro-angiogenic peptides using stable, disulfide-rich cyclic scaffolds. Blood 118:6709–6717. 22039263 10.1182/blood-2011-06-359141

[18] Kimura RH, Teed R, Hackel BJ, Pysz MA, Chuang CZ, et al (2012) Pharmacokinetically stabilized cystine knot peptides that bind alpha-v-beta-6 integrin with single-digit nanomolar affinities for detection of pancreatic cancer. Clin Cancer Res 18:839–849. 22173551 10.1158/1078-0432.CCR-11-1116 PMC3271184

[19] Aboye TL, Ha H, Majumder S, Christ F, Debyser Z, et al (2012) Design of a novel cyclotide-based CXCR4 antagonist with anti-human immunodeficiency virus (HIV)-1 activity. J Med Chem 55:10729–10734. 23151033 10.1021/jm301468k PMC3521869

[20] Mulvenna JP, Wang C, Craik DJ (2006) CyBase: a database of cyclic protein sequence and structure. Nucleic Acids Res 34:D192–194. 16381843 10.1093/nar/gkj005 PMC1347368

[21] Gunasekera S, Foley FM, Clark RJ, Sando L, Fabri LJ, et al (2008) Engineering stabilized vascular endothelial growth factor-A antagonists: synthesis, structural characterization, and bioactivity of grafted analogues of cyclotides. J Med Chem 51:7697–7704. 19053834 10.1021/jm800704e

[22] Getz JA, Rice JJ, Daugherty PS (2011) Protease-resistant peptide ligands from a knottin scaffold library. ACS Chem Biol 6:837–844. 21615106 10.1021/cb200039s PMC3158827

[23] Eliasen R, Daly NL, Wulff BS, Andresen TL, Conde-Frieboes KW, et al (2012) Design, synthesis, structural and functional characterization of novel melanocortin agonists based on the cyclotide kalata B1. J Biol Chem 287:40493–40501. 23012369 10.1074/jbc.M112.395442 PMC3504764

[24] Gran L (1973) On the effect of a polypeptide isolated from “Kalata-Kalata” (Oldenlandia affinis DC) on the oestrogen dominated uterus. Acta Pharmacol Toxicol (Copenh) 33:400–408. 4801084 10.1111/j.1600-0773.1973.tb01541.x

[25] Simonsen SM, Sando L, Rosengren KJ, Wang CK, Colgrave ML, et al (2008) Alanine scanning mutagenesis of the prototypic cyclotide reveals a cluster of residues essential for bioactivity. J Biol Chem 283:9805–9813. 18258598 10.1074/jbc.M709303200

[26] Huang YH, Colgrave ML, Clark RJ, Kotze AC, Craik DJ (2010) Lysine-scanning mutagenesis reveals an amendable face of the cyclotide kalata B1 for the optimization of nematocidal activity. J Biol Chem 285:10797–10805. 20103593 10.1074/jbc.M109.089854 PMC2856286

[27] Huang YH, Colgrave ML, Daly NL, Keleshian A, Martinac B, et al (2009) The Biological Activity of the Prototypic Cyclotide Kalata B1 Is Modulated by the Formation of Multimeric Pores. J Biol Chem 284:20699–20707. 19491108 10.1074/jbc.M109.003384 PMC2742835

[28] Wang CK, Wacklin HP, Craik DJ (2012) Cyclotides insert into lipid bilayers to form membrane pores and destabilize the membrane through hydrophobic and phosphoethanolamine-specific interactions. J Biol Chem 287:43884–43898. 23129773 10.1074/jbc.M112.421198 PMC3527971

[29] Nawae W, Hannongbua S, Ruengjitchatchawalya M (2014) Defining the membrane disruption mechanism of kalata B1 via coarse-grained molecular dynamics simulations. Sci Rep 4:3933. 24492660 10.1038/srep03933 PMC3910381

[30] Takada S (2012) Coarse-grained molecular simulations of large biomolecules. Curr Opin Struct Biol 22:130–137. 22365574 10.1016/j.sbi.2012.01.010

[31] Khalfa A, Tarek M (2010) On the Antibacterial Action of Cyclic Peptides: Insights from Coarse-Grained MD Simulations. J Phys Chem B 114:2676–2684. 20143883 10.1021/jp9064196

[32] Su Z-Y, Wang Y-T (2010) Coarse-Grained Molecular Dynamics Simulations of Cobra Cytotoxin A3 Interactions with a Lipid Bilayer: Penetration of Loops into Membranes. J Phys Chem B 115:796–802. 21192700 10.1021/jp107599v

[33] Gkeka P, Sarkisov L (2009) Interactions of Phospholipid Bilayers with Several Classes of Amphiphilic α-Helical Peptides: Insights from Coarse-Grained Molecular Dynamics Simulations. J Phys Chem B 114:826–839. 10.1021/jp908320b 20028006

[34] Zhao J, Zhao C, Liang G, Zhang M, Zheng J (2013) Engineering Antimicrobial Peptides with Improved Antimicrobial and Hemolytic Activities. J Chem Inf Model 53:3280–3296. 24279498 10.1021/ci400477e

[35] Kästner J (2011) Umbrella sampling. WIREs Comput Mol Sci 1:932–942.

[36] Wimley WC (2010) Describing the mechanism of antimicrobial peptide action with the interfacial activity model. ACS Chem Biol 5:905–917. 20698568 10.1021/cb1001558 PMC2955829

[37] Shenkarev ZO, Nadezhdin KD, Sobol VA, Sobol AG, Skjeldal L, et al (2006) Conformation and mode of membrane interaction in cyclotides. Spatial structure of kalata B1 bound to a dodecylphosphocholine micelle. FEBS J 273:2658–2672. 16817894 10.1111/j.1742-4658.2006.05282.x

[38] Rosengren KJ, Daly NL, Harvey PJ, Craik DJ (2013) The self-association of the cyclotide kalata B2 in solution is guided by hydrophobic interactions. Biopolymers 100:453–460. 23893463 10.1002/bip.22269

[39] Yau WM, Wimley WC, Gawrisch K, White SH (1998) The preference of tryptophan for membrane interfaces. Biochemistry 37:14713–14718. 9778346 10.1021/bi980809c

[40] de Jesus AJ, Allen TW (2013) The role of tryptophan side chains in membrane protein anchoring and hydrophobic mismatch. Biochim Biophys Acta 1828:864–876. 22989724 10.1016/j.bbamem.2012.09.009

[41] Kaas Q, Craik DJ (2010) Analysis and classification of circular proteins in CyBase. Biopolymers 94:584–591. 20564021 10.1002/bip.21424

[42] Van Der Spoel D, Lindahl E, Hess B, Groenhof G, Mark AE, et al (2005) GROMACS: fast, flexible, and free. J Comput Chem 26:1701–1718. 16211538 10.1002/jcc.20291

[43] Hess B, Kutzner C, van der Spoel D, Lindahl E (2008) GROMACS 4: Algorithms for Highly Efficient, Load-Balanced, and Scalable Molecular Simulations. J Chem Theory Comput 4:435–447. 26620784 10.1021/ct700301q

[44] Marrink SJ, de Vries AH, Mark AE (2003) Coarse Grained Model for Semiquantitative Lipid Simulations. J Phys Chem B 108:750–760.

[45] Marrink SJ, Risselada HJ, Yefimov S, Tieleman DP, de Vries AH (2007) The MARTINI force field: coarse grained model for biomolecular simulations. J Phys Chem B 111:7812–7824. 17569554 10.1021/jp071097f

[46] Monticelli L, Kandasamy SK, Periole X, Larson RG, Tieleman DP, et al (2008) The MARTINI Coarse-Grained Force Field: Extension to Proteins. J Chem Theory Comput 4:819–834. 26621095 10.1021/ct700324x

[47] Berendsen HJC, Postma JPM, van Gunsteren, WF, DiNola A, Haak JR (1984) Molecular dynamics with coupling to an external bath. J Chem Phys 81:3684–3690.

[48] Humphrey W, Dalke A, Schulten K (1996) VMD: visual molecular dynamics. J Molec Graphics 14: 33–38, 27–38. 10.1016/0263-7855(96)00018-5 8744570

[49] Rosengren KJ, Daly NL, Plan MR, Waine C, Craik DJ (2003) Twists, knots, and rings in proteins. Structural definition of the cyclotide framework. J Biol Chem 278:8606–8616. 12482868 10.1074/jbc.M211147200

[50] Risselada HJ, Marrink SJ (2008) The molecular face of lipid rafts in model membranes. Proc Natl Acad Sci USA 105:17367–17372. 18987307 10.1073/pnas.0807527105 PMC2579886

[51] Fantini J, Garmy N, Mahfoud R, Yahi N (2002) Lipid rafts: structure, function and role in HIV, Alzheimer’s and prion diseases. Expert Rev Mol Med 4:1–22. 10.1017/S1462399402005392 14987385

[52] Aloia RC, Tian H, Jensen FC (1993) Lipid composition and fluidity of the human immunodeficiency virus envelope and host cell plasma membranes. Proc Natl Acad Sci USA 90:5181–5185. 8389472 10.1073/pnas.90.11.5181 PMC46679

[53] Scott KA, Bond PJ, Ivetac A, Chetwynd AP, Khalid S, et al (2008) Coarse-Grained MD Simulations of Membrane Protein-Bilayer Self-Assembly. Structure 16:621–630. 18400182 10.1016/j.str.2008.01.014

[54] Kumar S, Rosenberg JM, Bouzida D, Swendsen RH, Kollman PA (1992) The weighted histogram analysis method for free-energy calculations on biomolecules. I. The method. J Comput Chem 13:1011–1021.

[55] Hub JS, de Groot BL, van der Spoel D (2010) g_wham–A Free Weighted Histogram Analysis Implementation Including Robust Error and Autocorrelation Estimates. J Chem Theory Comput 6:3713–3720.

