# Supplementary figures and images for: Dynamic Scenario of Membrane Binding Process of Kalata B1
Source: PLoS One. 2014 Dec 4;9(12):e114473. doi: 10.1371/journal.pone.0114473 (PMC4256454; doi:10.1371/journal.pone.0114473)

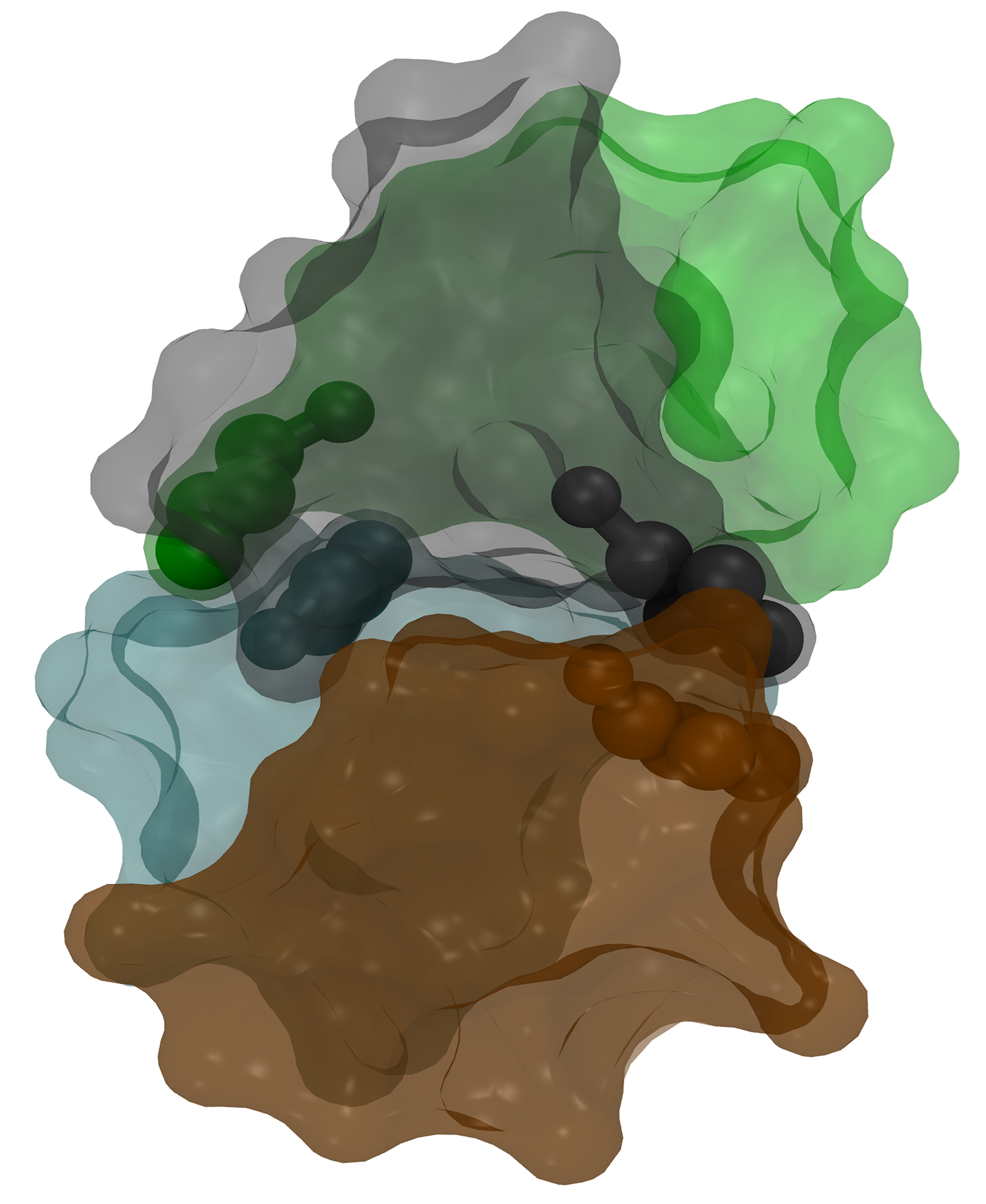

Supplement: Figure S1 — Pairing between side chains of Trp19 residues of kB1 tetramer in water. Molecules of kB1 are shown as transparent surface models. Molecules A–D are colored green, cyan, grey and orange, respectively. Trp19 is shown as a CPK model with the same color as the molecules. (TIF) [file pone.0114473.s001.tif]

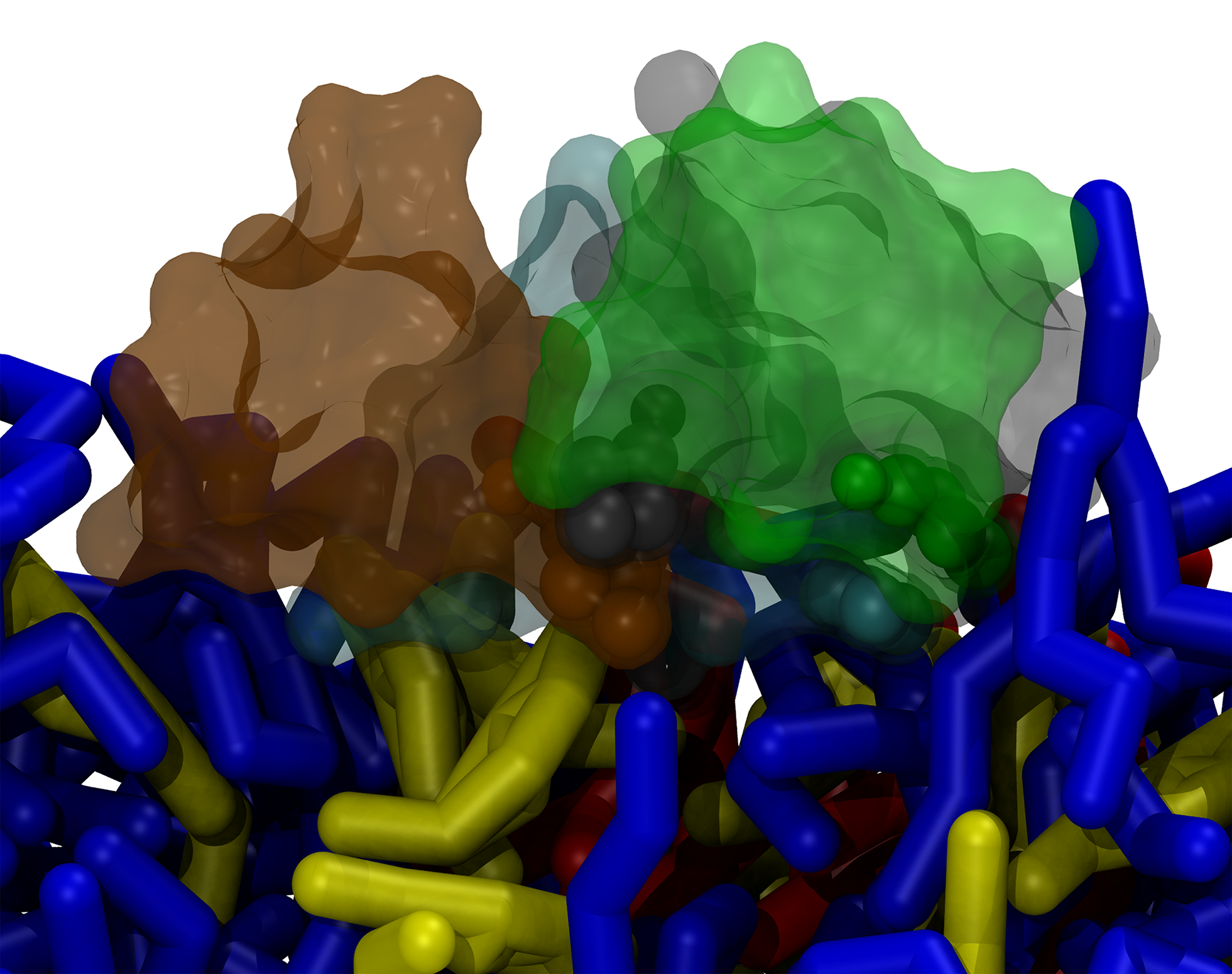

Supplement: Figure S2 — Pairing between side chains of Trp19 residues of kB1 tetramer in membrane-bound state. Molecules of kB1 are shown as transparent surface models. Molecules A–D are colored green, cyan, grey and orange, respectively. Trp19 is shown as a CPK model with the same color as the molecules. DUPC, DPPC and CHOL are represented as licorice models in blue, red and yellow, respectively. For clarity, water molecules are not shown. (TIF) [file pone.0114473.s002.tif]

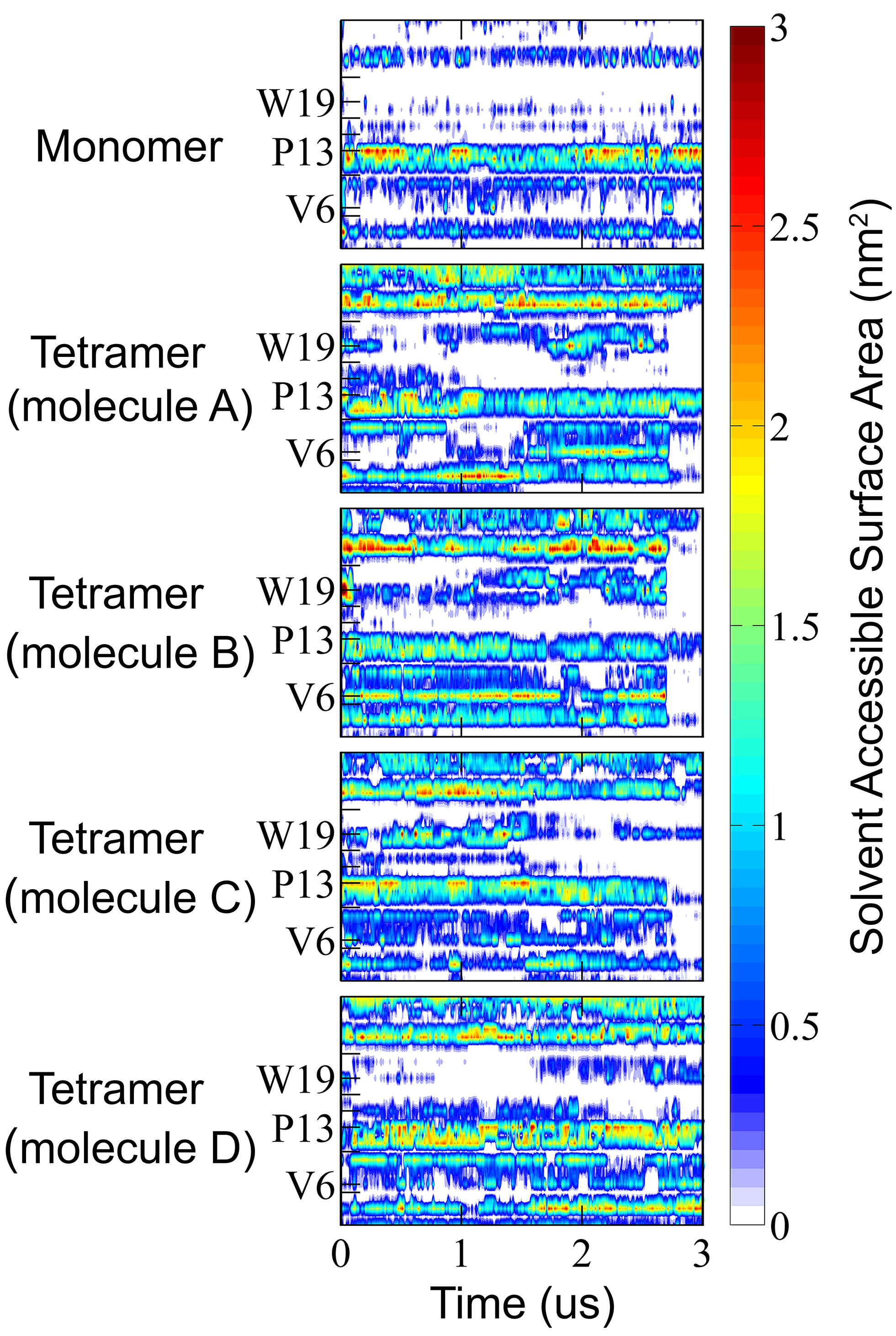

Supplement: Figure S3 — Exposure of AA residues to water. The solvent-accessible surface areas of each AA residue of the M1 monomer and all molecules in the tetramer are shown as a function of time across 1–3 µs. (TIF) [file pone.0114473.s003.tif]

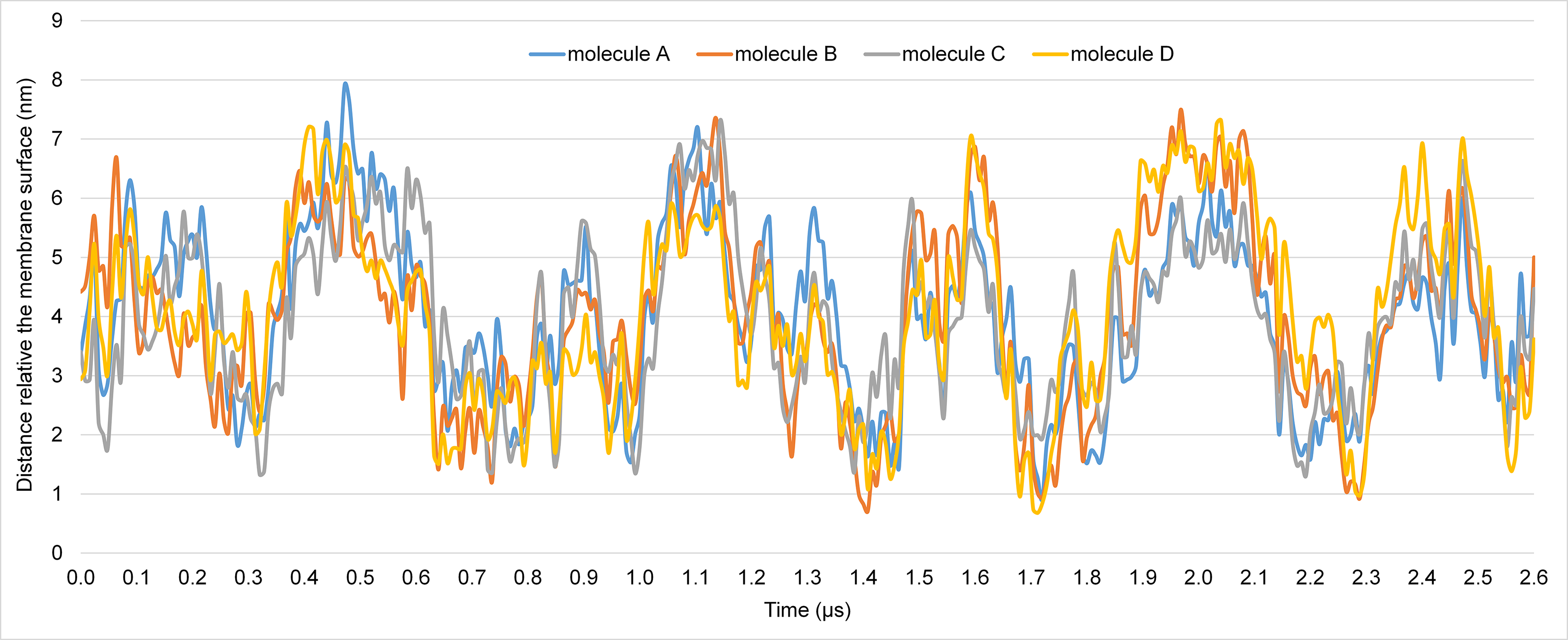

Supplement: Figure S4 — Approach of the tetramer to the membrane. The minimum distances of kB1 molecules in the tetramer relative to the membrane surface are shown. We assumed that the tetramer had approached the membrane when the distances were lower than 1.5 nm because at the same distance range the monomer can bind to the membrane. For instance, the distance to the membrane surface from the COM of Gly8 of molecule A at ca. 1.7 µs, Gly8 and Val6 of molecule B at ca. 1.4 µs and Glu3 and Gly7 of molecule D at ca. 1.7 µs was approximately 0.9, 0.7 and 0.7 nm, respectively. (TIF) [file pone.0114473.s004.tif]

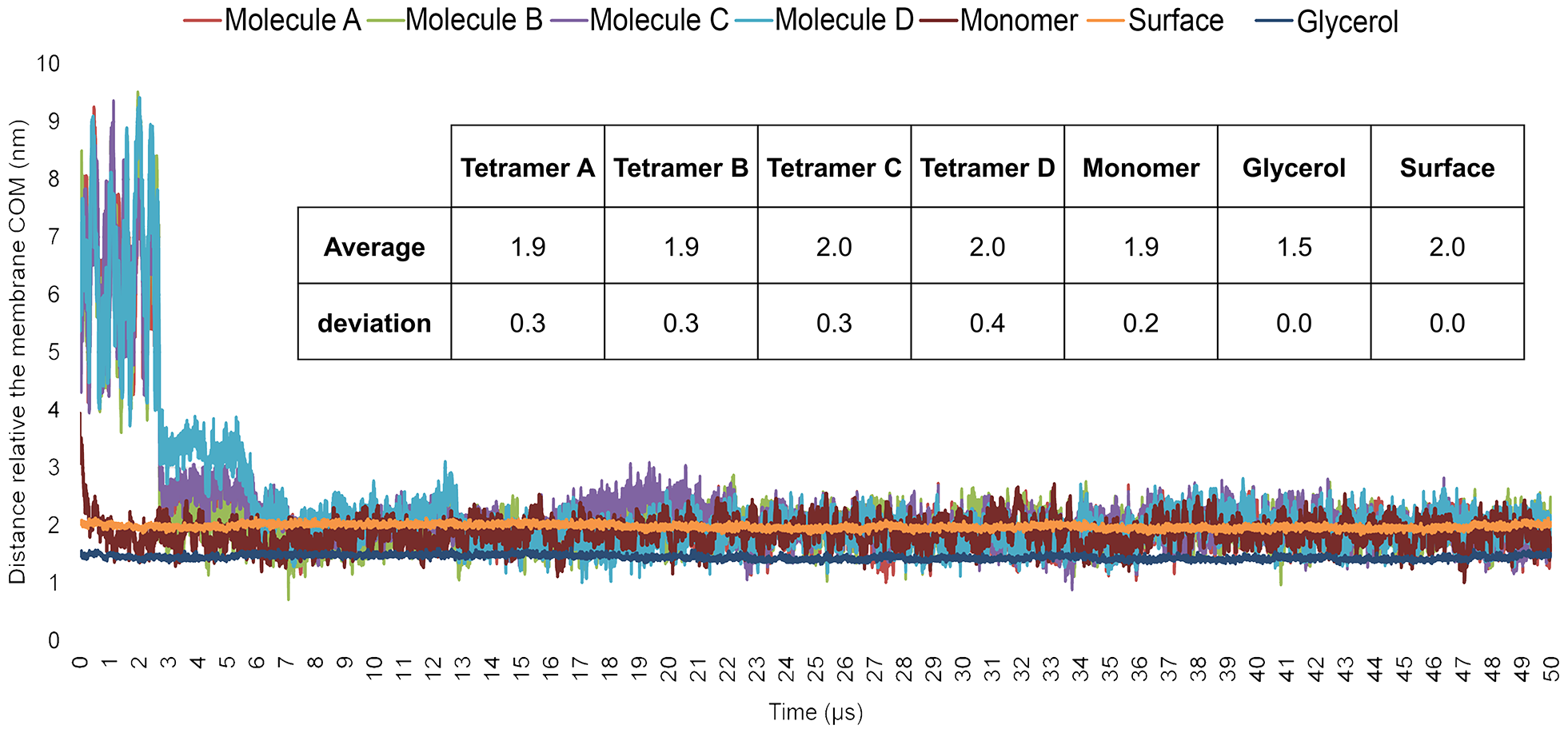

Supplement: Figure S5 — Position of kB1. Distances from the COM of monomeric kB1 (from the M1 simulations) and tetrameric kB1 to the COM of the membrane are shown as a function of time. Distances from the membrane surface and glycerol groups are also presented. The offset table shows the average distances and standard deviations from 23–50 µs. (TIF) [file pone.0114473.s005.tif]

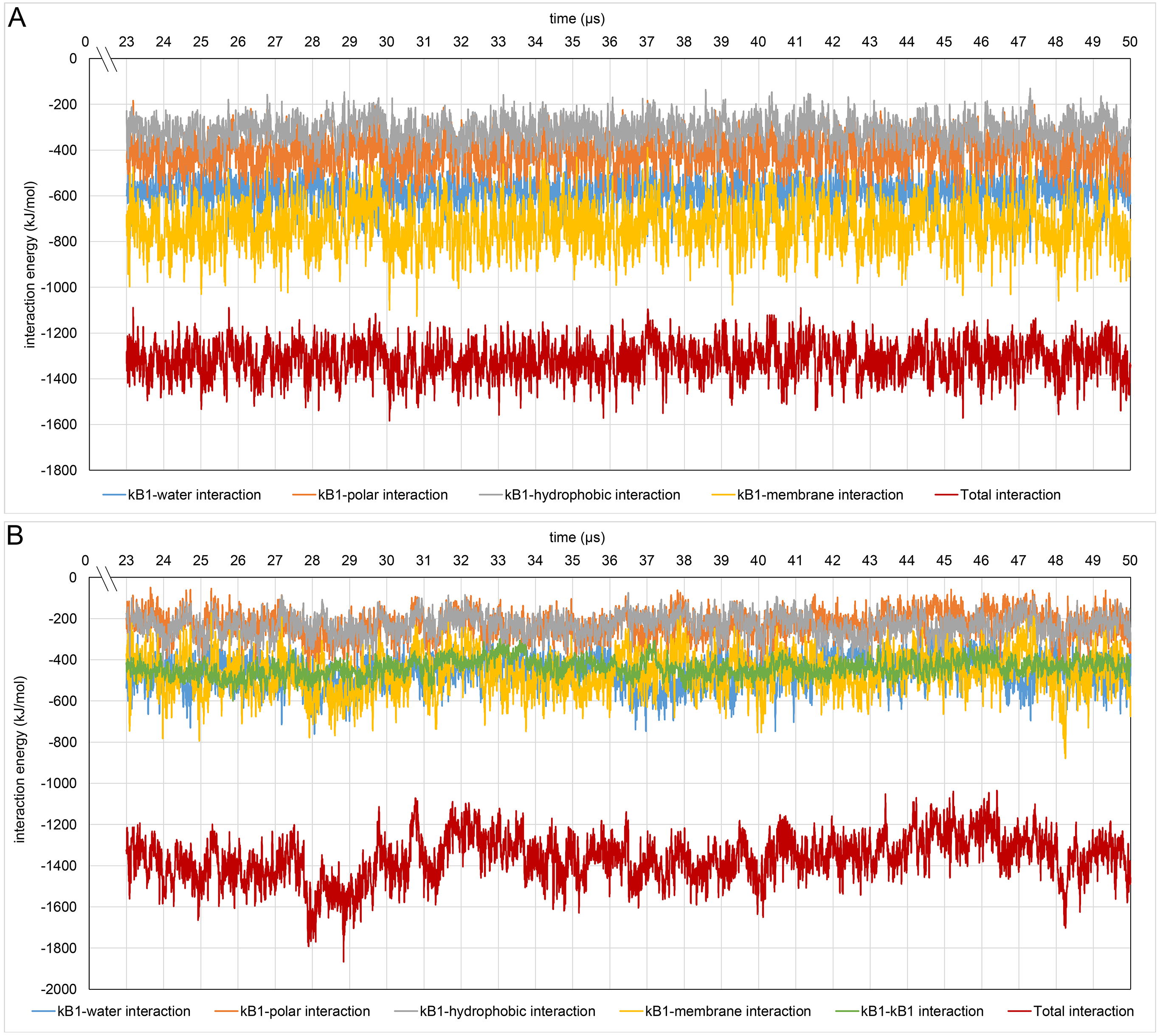

Supplement: Figure S6 — Interactions of kB1 at membrane-bound state. Interaction energies of (A) monomeric and (B) tetrameric kB1 as a function of time are shown respectively. (TIF) [file pone.0114473.s006.tif]

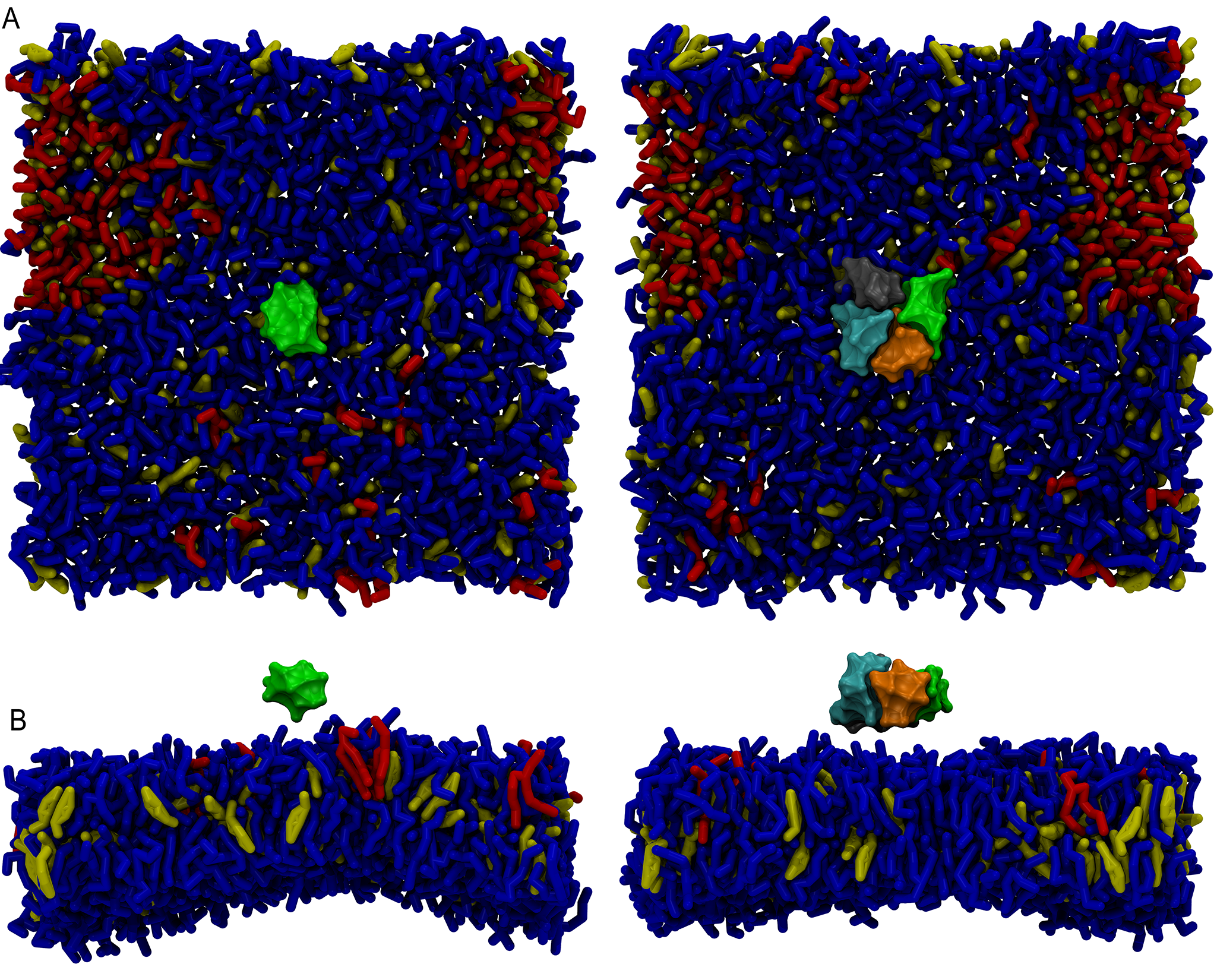

Supplement: Figure S7 — Configurations for pulling simulations. (A) Top view of the molecular coordinates selected from the monomer and the tetramer simulations for preparing initial configurations of the pulling simulations are shown. (B) Side view of initial configurations of the monomer and the tetramer used for PMF calculations are shown. DUPC, DPPC and CHOL are represented as licorice models in blue, red and yellow, respectively. The monomer is shown as a green surface model. Molecules A–D are shown as green, cyan, grey and orange surface models, respectively. Water is not shown for clarity. (TIF) [file pone.0114473.s007.tif]

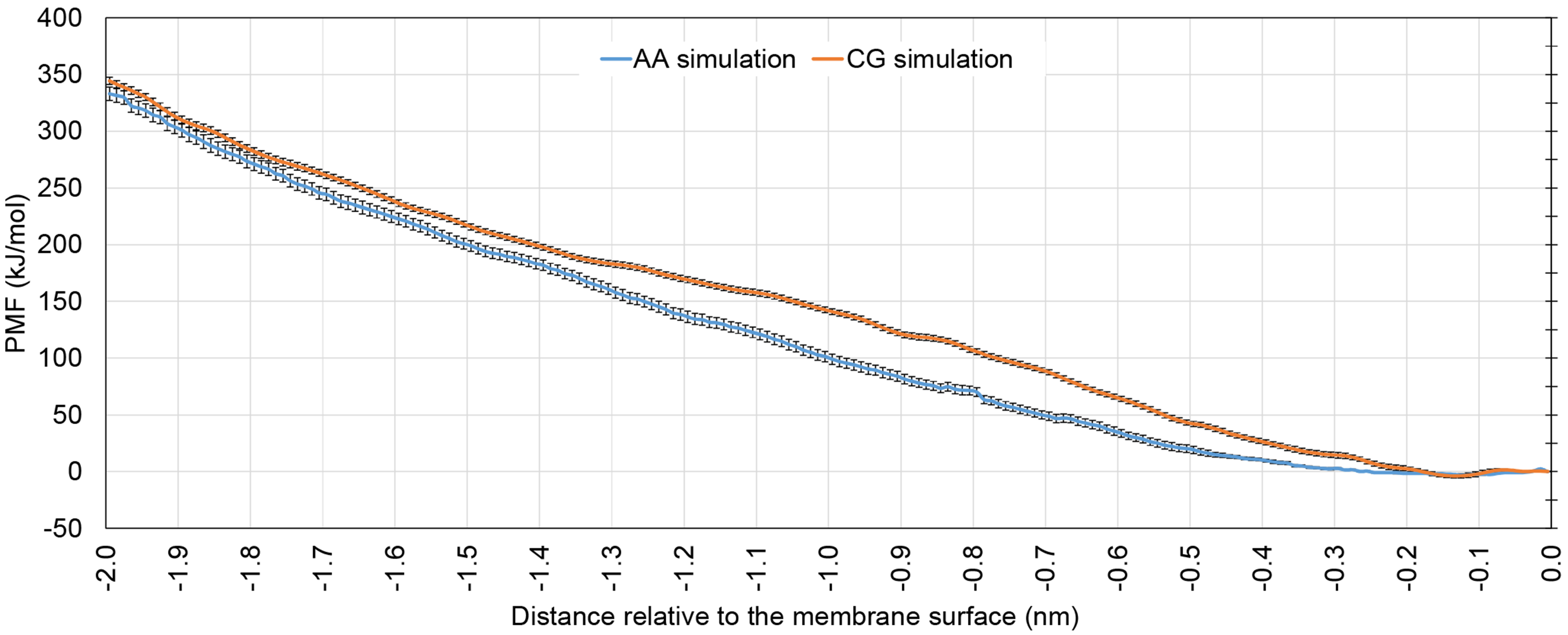

Supplement: Figure S8 — Validation of potentials of mean force profile. Mean force potentials for transferring atomistic (blue line) and coarse-grained (orange line) models of the monomer from the membrane surface (0.0 nm) to the COM of the membrane (−2.0 nm) are shown. Error bars were estimated using bootstrap analysis. (TIF) [file pone.0114473.s008.tif]

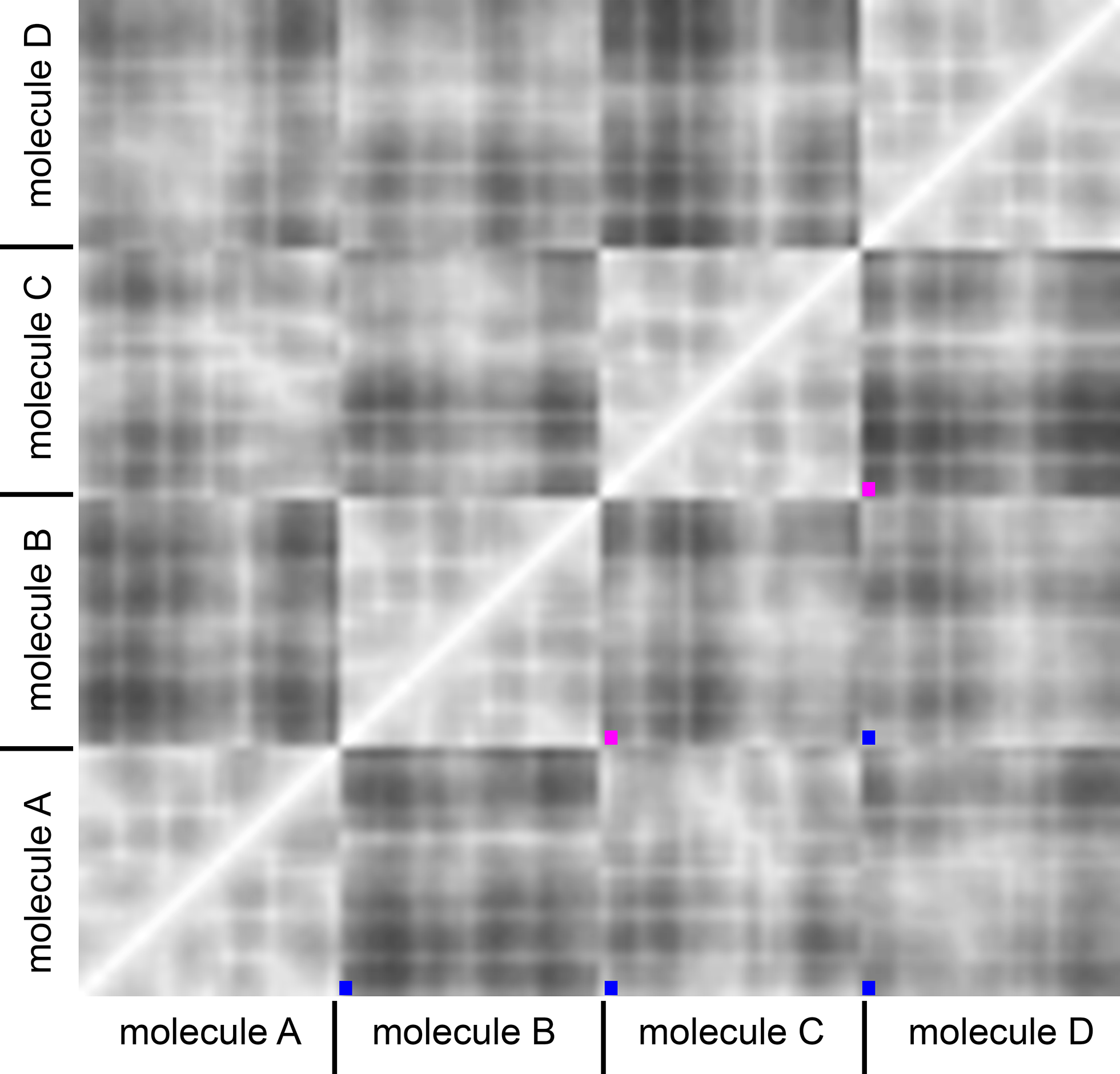

Supplement: Figure S9 — The minimum residue-to-residue distances matrix. The distances are shown in a grey scale where the shortest distance (0 nm) is shown as white. As described in the methods, magenta dots represent Cys1-Cys1 contact while blue dots represent Cys1-Cys1 non-contact. (TIF) [file pone.0114473.s009.tif]
