# Supplementary material for: Dynamic Scenario of Membrane Binding Process of Kalata B1
Source: PLoS One. 2014 Dec 4;9(12):e114473. doi: 10.1371/journal.pone.0114473 (PMC4256454; doi:10.1371/journal.pone.0114473)
Supplement: Table S1 — Details of the monomeric and tetrameric simulations systems. (PDF) [file pone.0114473.s010.pdf]

Table S1. Details of the monomeric and tetrameric simulation systems.

Simulation type	Number of DUPC[a]	Number of DPPC[b]	Number of CHOL[c]	Number of Water beads	Number of kB1s	Time (μs)
M1	620	67	313	24,000	1	10 and extend to 50
M2	620	67	313	24,000	1	10
M3	620	67	313	24,000	1	10
T1	620	67	313	24,000	4	20 and extend to 50
T2	620	67	313	24,000	4	20
T3	620	67	313	24,000	4	20

[a]diundecanoyl-phosphatidylcholine. [b]dipalmitoyl-phosphatidylcholine. [c]cholesterol.
