# Supplementary material for: Dynamic Scenario of Membrane Binding Process of Kalata B1
Source: PLoS One. 2014 Dec 4;9(12):e114473. doi: 10.1371/journal.pone.0114473 (PMC4256454; doi:10.1371/journal.pone.0114473)
Supplement: Table S2 — Average interaction energy of each AA residue of kB1 in the membrane-bound state. Interaction energies each AA residue with water and with the polar head and hydrophobic tail of the membrane were calculated in GROMACS program based on MARTINI CG force field. The reported interaction energies were extracted from the GROMACS energy files. Standard deviations of the average interaction energies are presented. (PDF) [file pone.0114473.s011.pdf]

Table S2. Average interaction energy of each AA residue of kB1 in the membrane-bound state.

AA residues of kB1	Monomer interaction (kJ/mol)			Tetramer interaction (kJ/mol)		
	Water	Polar	acyl-chain	Water	Polar	acyl-chain
C1	-24±9	-13±8	-3±5	-7±8	-9±8	-8±6
G2	-18±7	-10±7	-1±1	-8±7	-3±4	0±0
E3	-53±13	-21±12	-1±2	-40±14	-5±6	0±0
T4	-29±13	-23±13	-3±5	-35±12	-3±5	0±1
C5	-10±9	-18±8	-12±8	-21±11	-2±5	0±2
V6	-13±10	-20±10	-17±13	-28±10	-2±5	-1±3
G7	-14±6	-11±8	-1±2	-19±6	-1±3	0±0
G8	-24±8	-8±7	0±1	-26±9	-1±3	0±0
T9	-40±11	-7±8	0±1	-32±16	-4±8	0±1
C10	-12±7	-3±2	-1±2	-10±8	-1±3	0±1
N11	-52±12	-3±5	0±0	-45±14	-4±7	0±1
T12	-56±12	-3±5	0±1	-41±14	-9±13	-1±3
P13	-32±7	-2±4	-1±3	-13±8	-8±9	-8±12
G14	-16±5	-2±3	0±1	-4±4	-5±6	-2±4
C15	-15±7	-3±3	-1±3	-2±4	-7±7	-9±9
T16	-20±10	-9±8	-1±3	-8±7	-7±9	-3±5
C17	-6±5	-10±6	-6±6	-7±6	-4±5	-1±2
S18	-17±10	-30±11	-9±8	-10±9	-11±12	-3±6
W19	-14±12	-43±18	-61±21	-8±11	-21±19	-36±22
P20	-1±2	-7±5	-45±11	0±1	-6±5	-33±12
V21	-1±2	-7±5	-38±11	-1±2	-7±6	-27±12
C22	-1±2	-7±4	-9±7	-1±2	-4±5	-4±4
T23	-7±7	-22±10	-11±8	-6±7	-11±11	-5±7
R24	-46±16	-46±21	-5±5	-43±16	-28±24	-3±5
N25	-37±12	-28±12	-3±3	-28±12	-22±13	-2±3
G26	-6±4	-14±6	-4±4	-4±4	-11±7	-4±4
L27	-5±5	-16±7	-30±12	-3±4	-14±8	-28±12
P28	-5±5	-13±6	-32±11	-2±4	-10±6	-32±11
V29	-14±9	-18±8	-21±11	-7±9	-12±8	-23±12

Interaction energies each AA residue with water and with the polar head and hydrophobic tail of the membrane were calculated in GROMACS program based on MARTINI CG force field. The reported interaction energies were extracted from the GROMACS energy files. Standard deviations of the average interaction energies are presented.
